# High-Frequency Ultrasonography Imaging: Anatomical Measuring Site as Potential Clinical Marker for Early Identification of Breast Cancer-Related Lymphedema

**DOI:** 10.3390/biomedicines13061396

**Published:** 2025-06-06

**Authors:** Ivana Klarić-Kukuz, Danijela Budimir Mršić, Antonela Matana, Blaž Barun, Jure Aljinović, Maja Marinović-Guić, Ana Poljičanin

**Affiliations:** 1 Department of Health Studies, University of Split, 21000 Split, Croatia; ivanaklaric.k@gmail.com (I.K.-K.); danijelabudimir@gmail.com (D.B.M.); antonela.matana@gmail.com (A.M.); jaljinovic@gmail.com (J.A.); maja.marinovic.guic@gmail.com (M.M.-G.); 2 Department of Diagnostic and Interventional Radiology, University Hospital of Split, 21000 Split, Croatia; 3 School of Medicine, University of Split, 21000 Split, Croatia; 4 Institute of Physical Medicine and Rehabilitation with Rheumatology, University Hospital of Split, 21000 Split, Croatia; blaz.barun1@gmail.com

**Keywords:** lymphedema, breast cancer, diagnostic ultrasound, cross-sectional anatomy, disease early detection

## Abstract

**Background/Objectives**: Accurate diagnosis of breast cancer-related lymphedema remains a clinical challenge. This study evaluated the diagnostic utility of ultrasound in detecting early lymphedema compared to conventional criteria, including the International Society of Lymphology staging and limb volume measurements. **Methods**: In this retrospective cross-sectional study, 68 female participants with unilateral breast cancer, who had completed cancer treatment at least six months before study enrolment, underwent both ultrasonographic assessment and standard limb circumference measurements. Ultrasound was performed bilaterally at five standardized anatomical sites. Sonographic parameters included assessment of cutaneous and subcutaneous thickness and echogenicity. Clinical staging and symptom profiles were assessed using ISL criteria and a structured questionnaire. Volume status was determined by relative volume change (RVC). **Results**: Among 68 participants, 36 were classified as ISL stage 0 and 32 as ISL stage II, 30 had RVC < 5%, while 38 had RVC ≥ 5%. Advanced stages were associated with older age. Multivariate analysis identified increased skin thickness at the medial upper arm cutis as significantly correlated with RVC ≥ 5% (OR 1.49, 95% CI: 1.01–2.21, *p* = 0.047). A similar trend was observed at the medial forearm (OR 1.3 (95% CI: (0.99, 1.71))). **Conclusions**: This study highlights ultrasound’s potential for early breast cancer-related lymphedema detection, especially in patients with minimal volume changes where conventional methods fall short. Increased cutaneous thickness in the medial upper arm emerged as a sensitive marker of early disease, while subcutaneous thickness and echogenicity may reflect in advanced stages. This distinction underscores the clinical value of cutaneous thickness as a potential clinical key marker for early lymphedema detection, emphasizing the need for standardized protocols and defined thresholds to guide timely interventions.

## 1. Introduction

Breast cancer-related lymphedema (BCRL) commonly arises from lymphatic system impairment following lymph node dissection, other surgical intervention, or radiation therapy, resulting in disrupted lymphatic flow [1]. This impairment leads to the accumulation of lymphatic fluid, causing structural alterations in the skin and subcutaneous compartments [2]. Clinically, BCRL may manifest as progressive swelling of the arm, shoulder, neck, or torso, often accompanied by discomfort, tightness, and heaviness, ultimately contributing to functional impairment and diminished quality of life [1,3,4].

The true incidence of BCRL is challenging to ascertain due to variability in diagnostic methodologies, treatment modalities, and patient-specific factors [5,6,7]. The reported incidence of arm BCRL is approximately 21% [6,7]. Although current surveillance models emphasize early detection, BCRL often remains undiagnosed until pronounced swelling occurs [8,9]. Delayed diagnosis hampers early intervention, reducing treatment efficacy and adversely impacting patient functionality and quality of life [10,11,12,13,14].

Preventing BCRL progression is critical and necessitates timely intervention upon recognition of early signs [15,16,17,18]. Prevention strategies include primary prevention, aiming to avoid disease onset; secondary prevention, focusing on early-stage treatment to inhibit disease progression; and tertiary prevention, managing advanced-stage BCRL [19,20]. Despite the existence of prevention guidelines, they often rely on anecdotal recommendations lacking robust empirical support, leaving clinicians without proper prevention strategies [10]. The gold standard rehabilitation strategy of BCRL is complete decongestive therapy (CDT), which should be individually tailored depending on disease severity and includes compression therapy, manual lymphatic drainage, exercise, skin care, and patient education [11,12,13,15,17,18,19,20].

Accurate early diagnosis of BCRL remains challenging due to reliance on subjective clinical assessment, volumetric measurements, and patient-reported symptoms, which often lack precision and specificity [6,10,21]. The International Society of Lymphology (ISL) classifies subclinical BCRL primarily by patient’s perception of swelling [10,22]. Subjective assessments, such as palpation and patient-reported symptoms, often demonstrate inconsistencies, reducing diagnostic reliability [23,24]. Although circumferential and volumetric measurements are reliable for assessing total limb volume, including bones, muscles, fat, and other soft tissues, they lack specificity for BCRL [17,24,25,26]. Volume increases may reflect fluid accumulation or pathological tissue proliferation, which standard methods cannot distinguish, risking misinterpretation of underlying skin changes [10,13,23,27,28].

Advanced imaging modalities such as computed tomography, magnetic resonance imaging, lymphoscintigraphy, and indocyanine green lymphography provide more detailed visualization of lymphatic structures. Still, they are limited by high costs, invasiveness, and poor portability [19,24,29].

Effective BCRL management necessitates a simple, accessible diagnostic tool to confirm subjective findings at the earliest stage, enabling differentiation between subclinical and clinical BCRL. Emerging research suggests that high-resolution ultrasonography (US) may be a valuable tool for precise BCRL assessment and accurate management. However, it is not yet widely implemented in clinical practice [29,30,31,32,33,34,35,36,37,38,39].

Other non-invasive modalities, including bioimpedance spectroscopy (BIS) and tissue dielectric constant (TDC) measurements, provide advantages in cost, portability, and effective use but lack the anatomical resolution provided by US. While US requires more training and operator expertise, it provides superior visualization of subcutaneous tissues and is particularly valuable in ambiguous cases [40,41].

Previous US studies have primarily focused on advanced-stage BCRL, highlighting the correlation between subcutaneous echogenicity and disease severity [31,37]. Ultrasound effectively distinguishes various progression patterns by identifying variations in echogenicity and echo-free spaces, therefore providing valuable insights into edema and fibrosis in the skin and subcutaneous tissues and serving as a reliable method for monitoring treatment outcomes or disease progression over time [37,42,43].

Despite promising applications, standardized ultrasonographic protocol for assessing subclinical-stage BCRL remains underdeveloped, emphasizing the need for further research and clinical validation [3,31,33,34,35,36,37,38,39,44,45,46,47].

Current scarce literature reveals considerable methodological variability across studies of upper limb BCRL, including cross-sectional, diagnostic accuracy, longitudinal, prospective, and retrospective designs [2,3,4,31,35,45,47,48,49]. Variations also arise in participant selection, lymphedema definitions, staging, and post-breast cancer surgery duration [3,39,46]. Ultrasound modalities applied in research include elastography, high-frequency grayscale, and B-mode ultrasound, typically ranging from 11 to 18 MHz [2,3,4,31,35,45,47,48,49]. Measurement protocols differ in assessed parameters such as tissue thickness, echogenicity, cross-sectional area changes, and anatomical reference points, resulting in limited comparability [3,45,47]. Additionally, inconsistencies between absolute thickness measurements and relative indicators complicate standardization efforts [3,32,43,50,51]. Establishing uniform ultrasound protocols with clearly defined anatomical landmarks is critical to enhance clinical validity and comparability across studies [2,3].

This study aims to assess the effectiveness of US in early detection and management of lymphedema (LE) in breast cancer survivors by comparing its diagnostic reliability with conventional methods such as the ISL staging system and limb volume measurements. Specifically, our research seeks to determine whether US can identify subclinical LE changes before they become apparent through traditional assessments, facilitating earlier intervention and potentially improving patient outcomes. Additionally, the study evaluates the consistency and accuracy of US measurements relative to established diagnostic tools, aiming to develop standardized protocols for US integration into routine clinical practice.

## 2. Materials and Methods

### 2.1. Ethical Approval

The study protocol was approved by the Ethical Committee of the University Hospital Split (protocol code 2181-147/01/06/LJ.Z.-23-2). This study was conducted following the principles of the Declaration of Helsinki. Before enrolment in the study, all participating women were informed about the nature of the study, and written informed consent was obtained.

### 2.2. Study Population

**Figure 1:**
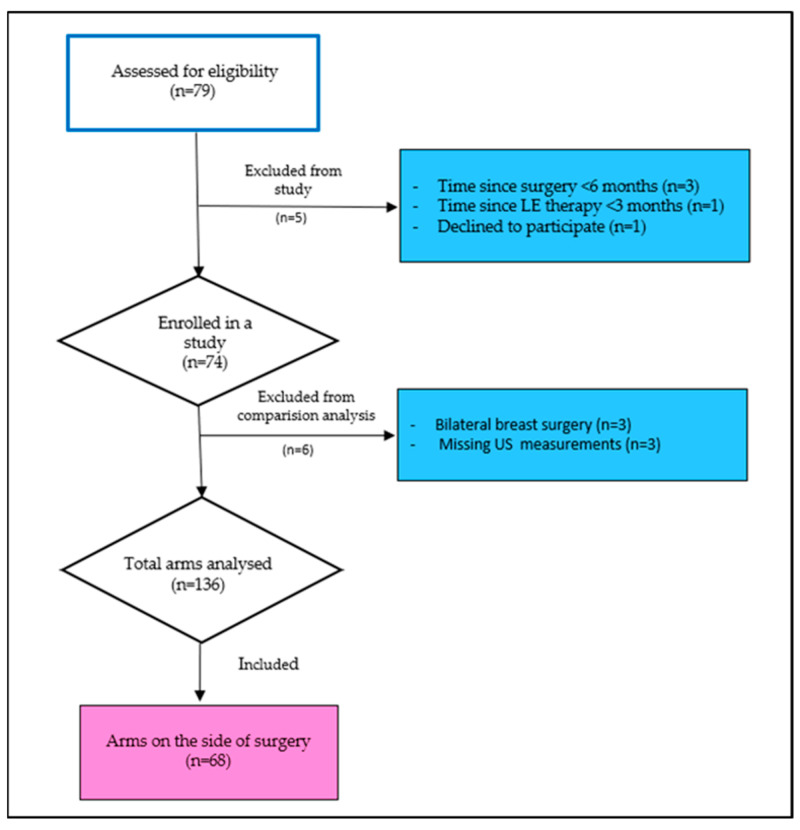
Flowchart of participants’ enrolment.

Seventy-nine breast cancer survivors participated in this cross-sectional observational study from 1 November 2023 to 30 April 2024. All breast cancer survivors, referred to the University Hospital Split Lymphedema Clinic during the study period, were consecutively screened for eligibility and invited to participate in the study. The eligibility criteria of the study population were women aged 18 years or older who had completed treatment for unilateral breast cancer at least six months before study enrolment. The exclusion criteria were women with bilateral or metastatic breast cancer, cognitive impairments, pre-existing arm lymphedema before the initiation of breast cancer treatment, and active lymphedema treatment within three months before enrolment. Following these exclusion criteria, data from 11 participants were excluded from the final analysis. Therefore, the final analysis was conducted on 68 breast cancer survivors (Figure 1).

### 2.3. Measurements and Data Collection

All participants underwent a single evaluation session at the University Hospital Split as part of this cross-sectional study. A trained researcher obtained baseline demographic data and a detailed medical history through structured clinical interviews and self-administered questionnaires. Missing data were addressed by reviewing participants’ electronic medical records. Table 1 summarizes the demographic and disease-related characteristics of the participants.

### 2.4. Lymphedema Assessment and Classification

#### 2.4.1. International Society of Lymphology (ISL) Classification

A physical medicine and rehabilitation specialist (A.P.) with expertise in lymphedema diagnosis and treatment conducted clinical staging of lymphedema according to ISL lymphedema severity staging criteria [10]. Subclinical lymphedema classified as Stage 0—included participants at risk of developing breast cancer lymphedema with lymph system obstruction due to the breast cancer treatment, with swelling not yet detectable but subtle tissue changes and self-reported symptoms possibly present. Clinical LE was classified as Stage I–III. In Stage I, reversible early fluid accumulation can be resolved with limb elevation. Stage II includes irreversible change, structural tissue changes, and moderate swelling, with early pitting or late-stage non-pitting edema due to fat and fibrotic changes. Stage III is characterized by severe swelling, trophic skin changes (e.g., acanthosis, fibrosis, fat deposits), and development of skin overgrowths [10,37].

#### 2.4.2. Limb Circumference Measurement

Limb circumference measurements were independently performed by two experienced and trained physiotherapists. To ensure consistency, two physiotherapists (I.K.K. and J.G.) jointly practiced the standardized measurement protocol before data collection to align their techniques and reduce potential variability in measurement methods. To evaluate inter- and intra-observer reliability, 20% of participants were randomly selected for repeated measurements by blinded physiotherapists concerning prior results to ensure objectivity. To ensure intra-rater reliability, one physiotherapist re-measured the same participants at a separate time point under the same conditions, again blinded to prior results. Additionally, inter-rater reliability was conducted by two physiotherapists who measured the same participants independently and separately, without previous knowledge of each other’s results. Intraclass Correlation Coefficients for both intra- and inter-rater reliability were excellent (ICC ≥ 0.99) except for the third measuring point with moderate inter-rater reliability (ICC ≥ 0.65). To perform adequate measurements, participants were seated with their arm resting on an adjustable hydraulic table with their shoulders flexed at approximately 90° and their forearm in pronation [23]. Five measurements were taken every 10 cm at predetermined points along the arm starting at the ulnar styloid process of the wrist (designated as point 0) using a flexible Juzo tape with 1 mm accuracy. Measurements were repeated three times at each point. Average values were documented on a standardized form [23,44]. In this study, we used the most commonly applied objective clinical diagnostic cut-off criteria, interlimb circumference measurement difference ≥ 2 cm taken at any single measuring site along the upper extremity [27,52].

#### 2.4.3. Derived Limb Volume Measurements

The absolute limb volumes of affected and unaffected arms were computed using the truncated cone formula across four segmental volumes (0–10 cm, 10–20 cm, 20–30 cm, and 30–40 cm), as described in the literature [53,54]. Limb volume ratio was derived as the relative volume change (RVC) between the affected and unaffected arms, normalized to the contralateral arm volume [26,55,56]. Clinical diagnosis was set at RVC 10%, which helped rule in BCRL, but values below RVC 10% could be used to rule out [26,52]. Since this study aimed to identify early BCRL, it was recommended to adjust diagnostic criteria to a low diagnostic volume threshold of RVC ≥ 5% [17,20,23,26,39,57]. In this study, we did a subgroup analysis to detect BCRL set at RVC ≥ 10% and RVC ≥ 5% volume diagnostic thresholds.

#### 2.4.4. Patient Self-Perceived Lymphedema

As in previous research, self-perceived lymphedema was considered if the participant answered positively to a question asked by the researcher, “At the moment, does your arm at the side of the surgery feel swollen?” [58,59,60].

Women who reported self-perceived lymphedema were further investigated about the presence of specific symptoms in their arm, such as tightness, burning, numbness, feeling of pins and needles, feeling of heaviness, feeling of tight clothes, feeling of tight jewellery, and pain sensation [58,59,60].

#### 2.4.5. Ultrasound Examination of Upper Limbs

Each participant underwent an ultrasound examination of the breast, axillary lymph nodes, and upper limbs during the evaluation session. Ultrasound measurements were performed using a high-frequency linear probe (9–12 MHz) on an ultrasound system, SuperSonic Imagine Aixplorer MACH 30, Aix-en-Provence, France. The affected upper limb was evaluated to assess the skin’s and subcutaneous tissue’s thickness and sonographic characteristics. For comparison, the contralateral, non-affected limb was also examined.

**Figure 2:**
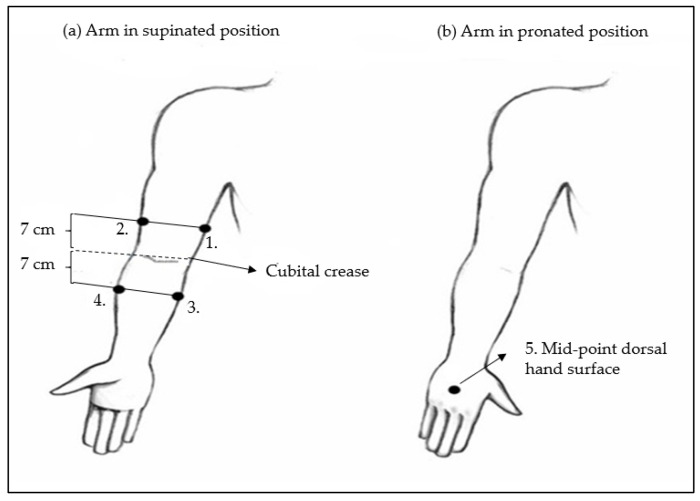
Schematic representation of ultrasound arm measuring points for cutis and subcutis thickness determination. (**a**) Arm positioned in supinated position: 1. Medial upper arm and 2. Lateral upper arm measuring points are placed 7 cm above the cubital crease. 3. Medial forearm and 4. Lateral forearm measuring points are placed 7 cm below the cubital crease. (**b**) Arm in pronated position: 5. Mid-point dorsal hand surface measuring point is placed between the wrist and the first metacarpophalangeal joint.

In the initial measurement position, participants were seated with their upper arm slightly abducted, elbow fully extended, and hand in supination. For the fifth measurement, participants placed their hands in a pronated position on the dorsal side of the hand. Measurement points were marked at five locations on each limb using a non-stretched centimetre tape, as described in the literature [15]: Upper arm medial and upper arm lateral, 7 cm above the cubital crease; medial and lateral forearm, 7 cm below the cubital crease; and dorsal hand surface, mid-point between the wrist and the first metacarpophalangeal joint. A total of 10 measurement sites per participant were assessed, 5 on each arm (Figure 2).

After applying a gel layer between the transducer and the skin, the transducer was placed at each site perpendicular to the skin with minimal pressure to avoid compressing the measured tissue layers. At each site, the thickness of the skin and subcutaneous tissue was recorded in centimetres and compared with the corresponding site on the contralateral arm. Subcutaneous tissue thickness was defined as the distance from the posterior echogenic border of the dermis to the anterior echogenic border of the deep muscular fascia. Additionally, the sonographic pattern of the subcutaneous tissue was assessed and classified as “normal”, “sclerotic”, “fluid”, or “no clear border between skin and subcutaneous tissue” according to Mander et al.’s proposed classification system [3].

All measurements were performed in the early morning to control for diurnal variation in skin water content. They were conducted by an experienced radiologist with over 10 years of expertise in ultrasound imaging who completed additional training specific to the study protocol.

For quality assurance, two radiologists (D.B.M. and M.G.M.) practiced a standardized measurement protocol before data collection. To formally assess measurement reliability, 10% of participants were randomly reassessed by radiologists blinded to the prior results. First, radiologists measured the same participants independently and separately for inter-rater reliability. Furthermore, to ensure intra-rater reliability, one radiologist remeasured the same participants at a separate time point under the same conditions. Most measurements indicated excellent or good agreement across nearly all measurement sites, specifically, upper arm lateral skin (ICC ≥ 86), upper lateral subcutis (ICC ≥ 0.91), upper arm medial cutis (ICC ≥ 0.91), upper arm medial subcutis (0.89), forearm lateral cutis (ICC ≥ 0.94), forearm lateral subcutis (ICC ≥ 0.81), forearm medial cutis (ICC ≥ 0.95), forearm medial subcutis (ICC ≥ 0.98), hand cutis (ICC ≥ 0.96), except inter-rater reliability for the fifth measuring point, which was slightly below good agreement (ICC ≥ 0.73)( Appendix A).

### 2.5. Sample Size

The sample size was estimated based on expected changes in skin and subcutaneous thickness [48]. At least 58 participants were required to detect the between-group difference, ensuring 80% power at a 5% significance level.

### 2.6. Statistical Analysis

The distribution of continuous variables was assessed using the one-sample Kolmogorov–Smirnov test. Variables with a normal distribution were reported as mean ± standard deviation (SD), while non-normally distributed variables were presented as median and interquartile range (IQR). Categorical variables were summarized as frequencies and percentages. As appropriate, differences between groups were evaluated using the chi-square test for categorical variables and either Student’s *t*-test or the Mann–Whitney U test for continuous variables.

We performed univariate analyses to investigate associations with the dependent variables (ISL stage, cm categories, and PEV categories). Variables that reached statistical significance (*p* < 0.05) were then included in multivariable models. Given the limited number of outcome events and the potential risk of overfitting, we applied least absolute shrinkage and selection operator (LASSO) regression with 10-fold cross-validation to select relevant predictors. All candidate variables were retained by LASSO (i.e., coefficients > 0), suggesting potential importance.

Subsequently, we fitted age-adjusted multivariable logistic regression models including the LASSO-selected predictors. Odds ratios (ORs) and 95% confidence intervals (CIs) were reported. To internally validate the models and assess coefficient stability, we applied nonparametric bootstrapping with 1000 resamples. Model discrimination was evaluated using the area under the receiver operating characteristic (ROC) curve (AUC), and model calibration was assessed using the Hosmer–Lemeshow goodness-of-fit test. All statistical analyses were performed using JASP (Version 0.18.3) and R. A *p*-value < 0.05 was considered statistically significant.

## 3. Results

### 3.1. Participants’ Demographics and Disease-Related Characteristics

This study analyzed a total of 68 breast cancer survivor participants’ data. The period elapsed since breast cancer surgery until study enrolment was on average five years. According to BCRL ISL classification, participants were distributed into two groups: ISL Stage 0 group (ISL 0) with 36 (52.9%) and ISL Stage II group (ISL II) with 32 (47.1%) participants. Furthermore, we divided participants into two groups according to RVC category < 5% or ≥5%. Study participants in these two groups were statistically similar, except for age (*p* < 0.001), where ISL II (61.63 ± 7.13) and RVC ≥ 5% (54.64 ± 8.15) participants tended to be older.

### 3.2. Participants’ Lymphedema Characteristics

The outcomes of LE characteristics, categorized according to the ISL stage classification, are summarized in Table 2. We analyzed the data from breast cancer participants according to their clinical stage of BCRL (ISL 0 and ISL II). We found a significant statistical difference between the groups for most BCRL characteristics (*p* < 0.001), except for pain in the affected arm (*p* = 0.666). Additionally, the outcomes of BCRL characteristics, based on volume change, are summarized in Table 3. When using the minimal detectable volume change to identify low volume LE, with a cut-off value of RVC ≥ 5%, we observed a significant statistical difference between the groups for most LE characteristics (*p* < 0.001), except for self-reported symptoms, erysipelas infections, and pain in the affected limb.

### 3.3. Factors Associated with Lymphedema Classified According to the International Society of Lymphology Classification

In the univariate analysis, significantly higher measurements were found at several points, specifically in the UaMC (*p* = 0.011) and UaMSc (*p* = 0.002) regions, as well as in the FaMC (*p* = 0.001) and FaMSc (*p* = 0.001) regions, in participants with ISL stage II LE. Additionally, increased echogenicity values were observed more frequently in individuals with ISL stage II LE (*p* = 0.001).

A multivariable logistic regression analysis was performed with the ISL stage as the dependent variable and UaMC, UaMSc, FaMC, FaMSc, and arm echogenicity as independent variables. The logistic regression model was statistically significant (*p* < 0.001, Nagelkerke R^2^ = 0.562, accuracy = 0.765). The multivariate analysis revealed statistical significance for two variables: the FaMC area (OR 1.71, 95% CI: 1.02–2.86, *p* = 0.041) and UaMSc (OR 1.05, 95% CI: 1.002–1.1, *p* = 0.041). Specifically, as the interlimb skin thickness increased in the FaMC and UaMSc regions, the likelihood of a higher ISL stage also increased (Table 4, Supplementary Materials: Figure S1)).

### 3.4. Factors Associated with Lymphedema Classified According to Relative Volume Change ≥ 10%

In the univariate analysis, significantly greater measurements at several points were identified, specifically UaMC (*p* = <0.001) and UaLC (*p* = 0.028) regions, as well as in FaMC (*p* = <0.001), FaMSc (*p* = 0.007), and FaLC (*p* = 0.009) regions in participants with RVC ≥ 10%. Moreover, increased echogenicity values were found more frequently in individuals with RVC ≥ 10% (*p* = 0.001).

A multivariable logistic regression analysis was conducted with RVC ≥ 10% as the dependent variable, and UaMs, UaMSc, FaMC, FaMSc, and arm echogenicity as independent variables. The logistic regression model was statistically significant (*p* < 0.001, Nagelkerke R^2^ = 0.575, accuracy = 0.843). A multivariate analysis model revealed statistical significance for only one variable: FaMS (OR 1.3 (95% CI: (0.99, 1.71), *p* = 0.063)). Specifically, as the interlimb skin thickness increased at the FaMC, the likelihood of an interlimb RVC ≥ 10% also increased (Table 5, Supplementary Materials: Figure S2).

### 3.5. Factors Associated with Lymphedema Classified According to Relative Volume Change ≥ 5%

In the univariate analysis, significantly greater measurements at several points were identified, specifically the UaMC (*p* = 0.014) and UaMSc (*p* = 0.032) regions, as well as at the FaMC (*p* = 0.007), FaFaMSc (*p* = 0.006), and FaFaLSc (*p* = 0.029) regions in participants with RVC ≥ 5% LE. Moreover, increased echogenicity values were found more frequently in individuals with RVC ≥ 5% LE (*p* = 0.007).

To reduce the risk of overfitting given the limited number of outcome events, LASSO regression with 10-fold cross-validation was used for variable selection. All candidate predictors (UaMC, UaMSc, FaLSc, FaMC, FaMSc, and arm echogenicity) were retained. These variables were then included in an age-adjusted multivariable logistic regression model with RVC ≥ 5% as the dependent variable. The model demonstrated good fit (Hosmer–Lemeshow *p* = 0.45), strong discriminative ability (AUC = 0.8), and explained a substantial proportion of variance (Nagelkerke R^2^ = 0.516). Internal validation using 1000 bootstrap resamples confirmed the robustness and stability of coefficient estimates.

In the final model, only one variable remained statistically significant: UaMC (OR = 1.49, 95% CI: 1.01–2.21, *p* = 0.047). Specifically, increased interlimb skin thickness at the UaMC region was associated with higher odds of exhibiting an interlimb volume difference ≥ 5% (Table 6, Supplementary Materials: Figure S3).

Due to sample size constraints, variables such as BMI, radiotherapy, and axillary surgery type, although clinically relevant, were not included in the multivariate model. However, to address potential concerns about confounding, we conducted a sensitivity analysis by adding each of these variables individually into the model. The results showed no meaningful changes in the odds ratios (ORs) or *p*-values, indicating that these factors did not substantially confound the observed associations. The results of this analysis are provided in the Supplementary Materials (Table S1).

## 4. Discussion

This study highlights the potential, simplicity, and clinical applicability of ultrasound-based cutaneous thickness measurements in the medial upper arm region as an effective tool for detecting subclinical BCRL. Based on the results of our study, we firmly believe that US has the potential to differentiate between patients who require early intervention and those suitable for preventive follow-up programs. Multivariate analysis enabled us to control potentially confounding variables and provided insights into the relative impact of individual clinical and ultrasound parameters.

A key finding of this study was a significant association between increased cutis thickness in the medial upper arm region and interlimb volume difference (OR 1.49 (95% CI: (1.01, 2.21), *p* = 0.047)), even when lower volume-based thresholds were considered (RVC ≥ 5%). Our research has highlighted the medial upper arm region as potentially significant, a critical anatomical site for early identification of tissue changes, with increased cutaneous thickness emerging as a particularly sensitive marker for early BCRL detection (Table 6). In contrast, subcutaneous thickness and echogenicity were less sensitive ultrasound measures for detecting early BCRL; they appear to be more indicative of advanced BCRL stages (Table 4 and Table 5) [32,43,51].

Furthermore, this distribution pattern aligns with patients’ subjective reports in previous research of mild fullness or heaviness in the upper arm, even in the absence of overt swelling [16,18,23,61,62]. Stout et al. proposed that the earliest signs of LE appear in superficial tissues adjacent to muscles, particularly in the forearm and distal upper arm, with localized soft tissue changes around the elbow preceding significant limb volume increases [14]. Another confirmation of our upper arm medial region finding (Table 6) aligns with anatomical studies indicating that the medial upper arm lymphatic pathway drains directly into axillary lymph nodes, which are commonly affected by surgical removal or radiation therapy [63,64]. Consequently, Friedman et al. described a fluid accumulation pattern predisposed to occur primarily in the posterior distal upper arm (triceps region) and the adjacent medial arm, designating it as a potentially important anatomical site for early BCRL detection [64]. We presume that the lack of statistical significance of lateral arm region measurements in this study lies in the fact that the lateral lymphatic pathways remain unaffected, as they bypass the axilla and serve as a compensatory route for lymphatic drainage following axillary lymph node dissection described by Johnson et al. [63].

Additionally, Johansson et al. detected tissue dielectric constant measurements of highly localized edema in the upper arm, even when total arm volume remained within normal limits [65]. The above-presented anatomical and volume-based studies give insight into possible BCRL location and are consistent with our results [14,62,63,64,65].

Our primary finding contrasts with scarce previous ultrasound studies, which did not identify the medial region of the upper arm as a significant site for BCRL detection. Instead, they focused on forearm anatomical sites [3,29,35,48,49,50]. Studies by Devoogdt et al. and Polat et al., for example, highlighted that changes in tissue thickness and stiffness of the forearm reflect latent lymphedema [46,48]. Our findings suggest that earlier studies may have overlooked early signs of LE by underestimating the proximal tissue changes pattern in BCRL patients by underestimating the pattern of proximal tissue changes in BCRL patients. However, across these studies, the overall sensitivity of US findings for subclinical lymphedema detection has been modest, and conclusions were inconsistent. The detection of early lymphedema tissue changes in the upper arm implies that BCRL may not universally begin in the forearm, as suggested in previous latent lymphedema US studies [48]. Instead, as indicated in our research, more proximal arm tissues could be involved earlier than previously believed.

We believe that the discrepancy in the identification of anatomical sites for early BCRL detection between our study and previous studies lies in the use of different BCRL diagnostic thresholds. The most commonly accepted volume criterion to diagnose BCRL still is RVC ≥ 10%, although it has already been demonstrated that this threshold may fail to capture subclinical or early BCRL cases, potentially classifying patients as not having BCRL, thereby delaying timely diagnosis and intervention that could prevent disease progression [13,19,23,27,28,39,62,66].

When a conservative, volume-based clinical definition of lymphedema was applied in our cohort, ultrasonography identified the medial forearm as a crucial anatomical site for BCRL identification. Specifically, using relative volume change ≥ 10% to define BCRL, we observed that a positive ultrasound finding at the medial forearm was associated with higher odds of lymphedema (OR 1.3, 95% CI 0.99–1.71, *p* = 0.063; see Table 5). Furthermore, we detected the same forearm measuring site when applying ISL stage classification (OR 1.71, 95% CI: 1.02–2.86, *p* = 0.041, see Table 4). Previous authors have also highlighted inconsistencies and limitations in detecting early BCRL using traditional diagnostic approaches [46,47,55].

The prominence of the medial forearm observed in our US findings among participants with more advanced lymphedema aligns with recent anatomical insights into lymphatic drainage patterns, which differ between the medial and lateral forearm regions [64]. Anatomical studies suggest variations in lateral lymphatic bundle length, which drains towards the deltoid-pectoral and supraclavicular lymph nodes, may leave the medial forearm without an adequate drainage route, leading to lymphatic fluid accumulation [63,64]. Additionally, the medial aspect of the forearm may be more prone to fluid stasis in advanced stages of BCRL due to gravitational effects [31,32].

In conclusion, our findings highlight the limitations of traditional volume-based criteria in detecting subclinical BCRL and underscore the value of ultrasound-based cutaneous thickness measurements as a sensitive and complementary tool for early diagnosis, enabling timely intervention and potentially preventing disease progression. Additionally, in the absence of a universally accepted gold standard for detecting subclinical BCRL, this study compared US to commonly used reference methods, ISL staging, and RVC thresholds of 5% and 10% [10,19,67]. These methods are imperfect reference standards, which may introduce bias and affect sensitivity and specificity estimates by misclassifying disease status, and thus distorting the true performance of ultrasound [68]. The 5% RVC threshold is increasingly favored, supported by expert consensus and evidence showing that early intervention at this level can prevent progression to chronic lymphedema [9,55,69]. This aligns with modern BCRL management’s preventive focus, whereas the more conservative 10% threshold may delay timely treatment [17,67]. Thus, the 5% RVC threshold is advocated as the primary benchmark for early detection.

Our study extends the current understanding of ultrasound’s diagnostic utility in BCRL by highlighting a previously under-recognized anatomical site, the medial upper arm, for early detection. It also reaffirms the importance of forearm assessments while providing new insights into how lymphedema might spread in the limb. These findings underscore the need for further investigation into the spatiotemporal development of BCRL, which could inform more effective surveillance strategies and tailored interventions for at-risk patients.

### 4.1. Limitations of the Study

A key limitation of our study is its cross-sectional design, which limits the ability to assess the temporal progression of BCRL and the predictive value of our ultrasound findings. Additionally, our study’s retrospective and single-centre nature may have introduced selection bias and limited the ability to determine causal inference. Future multicentre, prospective longitudinal studies should enrol breast cancer patients before the initiation of cancer treatment and follow them over time to evaluate whether subclinical changes in subcutaneous tissue thickness detected by ultrasound can predict the onset and progression of BCRL.

Since our institution is a tertiary referral centre, the diagnostic accuracy reported in this study may have been overestimated because of potential disease spectrum bias. However, our centre also serves as the sole facility within the county providing specialized lymphedema diagnosis and management. Consequently, all breast cancer survivors in the region requiring such services are routinely referred to our clinic. This dual role mitigates the risk of disease spectrum bias and enhances the generalizability of our findings to the broader population of breast cancer survivors in comparable healthcare settings. Additionally, the absence of preoperative baseline measurements restricted the ability to determine the exact magnitude of post-treatment changes or differentiate between pre-existing anatomical variations and LE-related alterations. However, this limitation was mitigated by including only patients with unilateral breast cancer, allowing the contralateral unaffected limb to serve as an internal control for each patient. The lack of follow-up assessments further limits the evaluation of the predictive value of ultrasonographic markers over time and their role in tracking disease progression.

Finally, although a 20 MHz frequency ultrasound probe offers greater sensitivity for superficial skin structures, we utilized a 9–12 MHz frequency probe to balance resolution with deeper tissue penetration. It may have limited detection of subtle dermal–epidermal changes but is more suitable for evaluating advanced lymphedema. We believe that the 9–12 MHz probe allowed consistent assessment of key dermal and subcutaneous features and reflects real-world constraints, supporting the applicability of our findings across diverse clinical settings.

### 4.2. Strengths of the Study

Compared to similar studies, this research included a relatively large sample size, enhancing statistical power and generalizability. Multiple measurements of cutaneous and subcutaneous tissue thickness and echogenicity across different anatomical regions provided a detailed and region-specific evaluation of LE-related changes. To ensure measurement reliability, ultrasonographic assessments demonstrated high inter- and intra-rater consistency, with all imaging performed by a single experienced radiologist blinded to surgical history, minimizing bias. Standardized protocols were also applied to circumference measurements, ensuring consistency in LE classification. Unlike studies relying solely on absolute volume differences, this research accounted for relative volume changes, such as weight fluctuations, improving diagnostic accuracy. Additionally, by employing multivariate analysis, this study simultaneously evaluated multiple factors, identifying independent predictors of LE progression while controlling for potential confounding variables. Integrating clinical and ultrasonographic parameters strengthened the precision and reliability of interpreting complex interactions related to LE development and progression.

Overall, this study’s strengths contribute to the robustness of our findings, while the acknowledged limitations highlight areas for future research to improve the diagnostic and prognostic utility of ultrasonography in BCRL assessment.

## 5. Conclusions

This is one of the most extensive studies performed, underscoring the potential of ultrasound as an effective tool for the early detection and monitoring of BCRL.

Among BCRL patients with low volume changes, conventional diagnostic methods used in clinical practice, such as ISL staging, circumference, and volume measurements, are often insufficiently sensitive to distinguish patients who require early intervention from those suitable for preventive follow-up programs. These limitations allow disease progression, significantly impacting patients’ physical and psychological well-being.

Our findings identified the medial upper arm region as a potentially important anatomical site for the early detection of BCRL-related tissue changes. This region has not been previously emphasized in early detection US studies. Specifically, increased cutaneous thickness in this area emerged as a sensitive marker for detecting early signs of the disease, even among participants with minimal limb volume changes. However, we recognize that this association, while statistically significant (OR 1.49), is modest, and the cross-sectional design precludes conclusions about causality. In contrast, subcutaneous thickness and echogenicity appeared less predictive of early-stage BCRL but may hold greater relevance in more advanced stages. This distinction underscores the clinical significance of cutaneous thickness measurements as a potential ultrasound parameter for timely identification and intervention before significant volume changes occur. Despite the promising diagnostic utility of ultrasound, the lack of standardized imaging protocols and echogenicity grading remains a challenge. Future research should employ longitudinal designs to validate predictive values of ultrasonographic findings and to monitor temporal changes in tissue characteristics associated with BCRL progression. Additionally, efforts should focus on developing and validating standardized US protocols and establishing threshold values for early detection to improve clinical decision making and optimize patient care.

## Figures and Tables

**Table 1:** Demographics and disease-related characteristics of study participants.

Variable	Lymphedema Stage		*p*-Value	Lymphedema RVC ^6^		*p*-Value
	ISL ^5^ 0 (*n* = 36)	ISL ^5^ 2 (*n* = 32)		RVC ^6^ < 5% (*n* = 30)	RVC ^6^ ≥ 5% (*n* = 38)	
Age, mean (SD)	54.64 ± 8.15	61.63 ± 7.13	<0.001	54.77 ± 7.8	60.7 ± 8.2	0.003
BMI ^1^, median (IQR)	26.35 (8.03)	27.1 (4.5)	0.551	26.9 (7.3)	27.1 (5.5)	0.780
Dominant is affected limb, n (%)	22 (61.1%)	20 (62.5%)	0.906	17 (56.7%)	25 (65.8%)	0.442
Time since BC ^2^ surgery, median (IQR)	4.5 (6.0)	5.0 (9.0)	0.558	4.0 (5.0)	5.0 (7.8)	0.857
Type of surgery, n (%)			0.167			0.081
Mastectomy	25 (69.4%)	17 (53.1%)		22 (73.3%)	20 (52.6%)	
Breast-conserving surgery	11 (30.6%)	15 (46.9%)		8 (26.7%)	18 (47.4%)	
Type of lymph node removal, n (%)			0.058			0.988
SLND ^3^	17 (47.2%)	8 (25.0%)		11 (36.7%)	14 (36.8%)	
ALND ^4^	19 (52.7%)	24 (75.0%)		19 (63.3%)	26 (68.4%)	
Post-surgery complications, n (%)	26 (72.2%)	22 (68.8%)	0.754	20 (66.7%)	28 (73.7%)	0.528
Axillary web syndrome n (%)	9 (25%)	6 (18.8%)	0.535	8 (26.7%)	7 (18.4%)	0.416
Radiotherapy application, n (%)	23 (63.9%)	26 (81.3%)	0.111	21 (70%)	28 (73.7%)	0.737
Chemotherapy application, n (%)	19 (52.8%)	19 (59.4%)	0.584	14 (46.7%)	24 (63.2%)	0.174
Type of chemotherapy application, n (%)		0.935			0.950	
Adjuvant	16 (44.4%)	15 (46.9%)		12 (40%)	19 (50.0%)	
Neoadjuvant	4 (11.1%)	4 (12.5%)		1 (3.3%)	3 (7.9%)	
Anti HER2 application, n (%)	7 (19.4%)	5 (15.6%)	0.771	6 (20.0%)	6 (15.8%)	0.383
Endocrine treatment, n (%)	27 (8%)	23 (71.9%)	0.771	25 (83.3%)	25 (65.8%)	0.103

^1^ Body mass index, ^2^ breast cancer, ^3^ sentinel lymph node dissection, ^4^ axillary lymph node dissection, ^5^ International Society of Lymphology classification, ^6^ relative volume change.

**Table 2:** Participants’ LE characteristics according to ISL stage classification.

Variable	ISL ^1^ 0 (*n* = 36)	ISL ^1^ II (*n* = 32)	*p*-Value
Increase of interlimb edema volume ratio, n (%)			<0.001
0–5%	27 (75%)	3 (9.4%)	
>5%	9 (25%)	29 (90.6%)	
Increase of interlimb edema volume ratio, n (%)			<0.001
0–10%	35 (97.2%)	14 (43.8%)	
>10%	1	18	
Interlimb circumference difference, n (%)			<0.001
<2cm	33 (91.6%)	6 (18.8%)	
≥2 cm	3 (8.3%)	26 (81.3%)	
Self-reported swelling, n (%)	13 (36.1%)	25 (78.1%)	<0.001
Self-reported LE symptoms, n (%)	20 (55.6%)	28 (87.5%)	0.004
Lymphedema complications, n (%):			
Pain in the LE ^2^ affected arm	15 (41.7%)	15 (46.9%)	0.666
Erysipelas episodes	1 (2.8%)	7 (21.9%)	0.015

^1^ International Society of Lymphology classification, ^2^ lymphedema.

**Table 3:** Participants’ LE characteristics according to volume ≥ 5%.

Variable	RVC^2^ < 5% (*n* = 30)	RVC^2^ ≥ 5% (*n* = 38)	*p*-Value
ISL stage			<0.001
^1^ ISL 0	27 (90%)	9 (23.7%)	
ISL II	3 (10%)	29 (76.3%)	
Interlimb circumference difference, n (%)			<0.001
<2 cm	28 (93.3%)	11 (29%)	
≥2 cm	2 (6.7%)	27 (71.1%)	
Self-reported swelling, n (%)	12 (40%)	26 (68.4%)	0.019
^2^ RVC			<0.001
<10%	30 (100%)	19 (50%)	
≥10%	0 (0%)	19 (50%)	
Self-reported ^3^ LE symptoms, n (%)	18 (60%)	30 (78.9%)	0.055
Lymphedema complications, n (%)			
Pain in LE LE-affected arm	13 (43.3%)	17 (44.7%)	0.908
Erysipelas episodes	1 (3.3%)	7 (18.4%)	0.055

^1^ International Society of Lymphology classification, ^2^ relative volume change, ^3^ lymphedema.

**Table 4:** Multivariate logistic regression analysis for the prediction of ISL stage.

	ISL Classification ^11^		Univariate Model	Multivariable Model	
Variable	ISL = 0 ^12^	ISL = II ^13^	*p*-Value	OR ^14^ (95% C.I)	*p*-Value
UaLC ^1^, median (IQR)	2.0 (2.5)	3.0 (4.0)	0.061		
UaLSc ^2^, median (IQR)	11.5 (16.25)	10.5 (19.25)	0.535		
UaMC ^3^, median (IQR)	1.5 (1.5)	3.0 (5.0)	0.011	1.32 (0.90, 1.95)	0.158
UaMSc ^4^, median (IQR)	14.0 (14.25)	27.0 (32.25)	0.002	1.05 (1.002, 1.1)	0.041
FaLC ^5^, median (IQR)	2.0 (1.0)	1.5 (3.0)	0.342		
FaLSc ^6^, median (IQR)	6.0 (7.25)	6.5 (9.5)	0.805		
FaMC ^7^, median (IQR)	1.0 (1.0)	2.5(6.25)	0.001	1.71 (1.02, 2.86)	0.041
FaMSc ^8^, median (IQR)	5.5 (7.5)	10.0 (22.25)	0.001	0.98 (0.93, 1.03)	0.431
HC ^9^, median (IQR)	12.5 (7.0)	12.5 (9.0)	0.961		
HSc ^10^, median (IQR)	2.0 (2.25)	2.0 (3.5)	0.737		
Arm echogenity n (%)			<0.001		0.272
Increased echogenicity	5 (22.7%)	17 (77.3%)		reference	
Normal echogenity	31 (67.4%)	15 (32.6%)		0.40 (0.08, 2.06)	

^1^ Upper arm lateral cutis, ^2^ upper arm lateral subcutis, ^3^ upper arm medial cutis, ^4^ upper arm medial subcutis, ^5^ forearm lateral cutis, ^6^ forearm lateral subcutis, ^7^ forearm medial cutis, ^8^ forearm medial subcutis, ^9^ hand cutis, ^10^ hand subcutis, ^11^ International Society of Lymphology classification, ^12^ International Society of Lymphology classification Stage 0, ^13^ International Society of Lymphology classification system, ^14^ odds ratios.

**Table 5:** Factors associated with lymphedema are classified according to interlimb relative volume change ≥ 10%.

	RVC % ^11^		Univariate Model	Multivariable Model	
Variable	<10%	≥10%	*p*-Value	OR (95% C.I)	*p*-Value
UaLC ^1^, median (IQR)	2.0 (3.0)	3.0 (10.0)	0.028	1.01 (0.93, 1.09)	0.909
UaLSc ^2^, median (IQR)	9.0 (17.0)	13.0 (22.0)	0.083		
UaMC ^3^, median (IQR)	2.0 (2.0)	4.0 (4.0)	<0.001	1.14 (0.88, 1.48)	0.316
UaMSc ^4^, median (IQR)	18.0 (19.0)	24.0 (44.0)	0.211		
FaLC ^5^, median (IQR)	1.0 (1.0)	2.0 (4.0)	0.009	1.42 (0.86, 2.34)	0.175
FaLSc ^6^, median (IQR)	6.0 (8.0)	7.0 (15.0)	0.215		
FaMC ^7^, median (IQR)	1.0 (1.0)	7.0 (8.0)	<0.001	1.3 (0.99, 1.71)	0.064
FaMSc ^8^, median (IQR)	6.0 (9.0)	13.0 (45.0)	0.007	1.02 (0.97, 1.07)	0.383
HC ^9^, median (IQR)	13.0 (7.0)	12.0 (9.0)	0.158		
HSc ^10^, median (IQR)	2.0 (3.0)	3.0 (8.0)	0.534		
Arm echogenicity category n (%)			<0.001		0.856
Increased echogenicity	10 (20.4%)	14 (66.7%)		reference	
Normal echogenitechogenicity	39 (79.6%)	7 (33.3%)		0.851 (0.15, 4.84)	

^1^ Upper arm lateral skin, ^2^ upper arm lateral subcutis, ^3^ upper arm medial skin, ^4^ upper arm medial subcutis, ^5^ forearm lateral skin, ^6^ forearm lateral subcutis, ^7^ forearm medial skin, ^8^ forearm medial subcutis, ^9^ hand skin, ^10^ hand subcutis, ^11^ relative volume change.

**Table 6:** Multivariate logistic regression analysis for the prediction of lymphedema relative volume change ≥ 5%.

	RVC % ^11^		Univariate Model	Multivariable Model	
Variable	<5%	≥5%	*p*-Value	OR (95% C.I)	*p*-Value
UaLC ^1^, median (IQR)	2.0 (2.75)	3.0 (4.0)	0.174		
UaLSc ^2^, median (IQR)	11.5 (16.75)	11.0 (19.0)	0.409		
UaMC ^3^, median (IQR)	1.5 (1.0)	3.0 (4.25)	0.014	1.49 (1.01, 2.21)	0.047
UaMSc ^4^, median (IQR)	14.0 (14.75)	22.0 (36.25)	0.032	1.01 (0.98, 1.05)	0.408
FaLC ^5^, median (IQR)	2.0 (1.0)	1.5 (3.0)	0.578		
FaLSc ^6^, median (IQR)	5.0 (7.75)	7.0 (12.0)	0.029	1.09 (0.99, 1.21)	0.087
FaMC ^7^, median (IQR)	1.0(1.0)	2.0 (6.25)	0.007	1.23 (0.87, 1.73)	0.238
FaMSc ^8^, median (IQR)	5.0 (5.75)	12.5 (31.0)	0.006	1.03 (0.97, 1.19)	0.329
HC ^9^, median (IQR)	12.0 (7.0)	13.0 (9.0)	0.934		
HSc ^10^, median (IQR)	2.0 (2.0)	2.0 (3.75)	1		
Arm echogenicity category n (%)			0.007		0.673
Increased echogenicity	5 (20.8%)	19 (79.2%)		Reference	
Normal echogenicity	25 (54.3%)	21 (45.7%)		0.71 (0.15, 3.48)	

^1^ Upper arm lateral skin, ^2^ upper arm lateral subcutis, ^3^ upper arm medial skin, ^4^ upper arm medial subcutis, ^5^ forearm lateral skin, ^6^ forearm lateral subcutis, ^7^ forearm medial skin, ^8^ forearm medial subcutis, ^9^ hand skin, ^10^ hand subcutis, ^11^ relative volume change.

## Data Availability

The data presented in this study are available on request from the corresponding author.

## Supplementary Materials

The following supporting information can be downloaded at: https://www.mdpi.com/article/10.3390/biomedicines13061396/s1, Figure S1: Forest plot showing summary results (Odds ratios (OR) and 95% confidence intervals (95% C.I) of multivariable regression analysis for the prediction of ISL stage. Multivariable model analysis revealed statistical significance for two variables: UaMSc and FaMC measuring points (*UaMC upper arm medial cutis, *UaMSc upper arm medial subcutis, *FaMC forearm medial cutis, *FaMSc forearm medial subcutis). Specifically, as the interlimb skin thickness increased in the FaMC and UaMSc regions, the likelihood of a higher ISL stage also increased (Table 4); Figure S2: Forest plot showing summary results (Odds ratios (OR) and 95% confidence intervals (95% C.I) of multivariable regression analysis for the prediction of RVC 10%. Multivariable model analysis revealed statistical significance for one variable: FaMC measuring point (*UaMC upper arm medial cutis, *UaMSc upper arm medial subcutis, *FaMC forearm medial cutis, *FaMSc forearm medial subcutis). Specifically, as the interlimb skin thickness increased in the FaMC region, the likelihood of RVC 10% also increased (Table 5). Figure S3: Forest plot showing summary results (Odds ratios (OR) and 95% confidence intervals (95% C.I) of multivariable regression analysis for the prediction of RVC 5%. Multivariable model analysis revealed statistical significance for one variable: UaMC measuring point (*UaMC upper arm medial cutis, *UaMSc upper arm medial subcutis, *FaMC forearm medial cutis, *FaMSc forearm medial subcutis). Specifically, as the interlimb skin thickness increased in the UaMC region, the likelihood of RVC 5% also increased (Table 6). Table S1: Results of the sensitivity analysis for the multivariate logistic regression analysis for the prediction of lymphedema relative volume change ≥ 5%. We included each of the variables—BMI, radiotherapy, and axillary surgery type—individually in the model

## Abbreviations

The following abbreviations are used in this manuscript:

LE	Lymphedema
BCRL	Breast cancer-related lymphedema
ISL	International Society of Lymphology
US	Ultrasonography
RVC	Relative volume change
ICC	Interclass correlation coefficient
SD	Standard deviation
IQR	Interquartile range
CI	Confidence intervals
UaLC	Upper arm lateral cutis
UaLSc	Upper arm lateral subcutis
UaMC	Upper arm medial cutis
UaMSc	Upper arm medial subcutis
FaLC	Forearm lateral cutis
FaLSc	Forearm lateral subcutis
FaMC	Forearm medial cutis
FaMSc	Forearm medial subcutis
HC	Hand cutis
HSc	Hand subcutis
ALND	Axillary lymph node dissection
MHz	Megahertz

## Appendix A

**Table A1:** Ultrasonography cutis and subcutis thickness reliability testing.

Measuring Site	ICC (95% C.I.)	ICC (95% C.I.)
	Inter-Rater Reliability Researcher 1 and Researcher 2	Inter-Rater Reliability Researcher 1 and Researcher 2
UaLC ^1^	0.890 (0.75, 0.96)	0.859 (0.68, 0.94)
UaLSc ^2^	0.870 (0.49, 0.97)	0.91 (0.79, 0.96)
UaMC ^3^	0.848 (0.66, 0.93)	0.910 (0.79, 0.96)
UaMSc ^4^	0.997 (0.99, 0.99)	0.889 (0.746, 0.954)
FaLC ^5^	0.963 (0.91, 0.99)	0.935 (0.85, 0,97)
FaLSc ^6^	0.830 (0.63, 0.93)	0.814 (0.60, 0.92)
FaMC ^7^	0.935 (0.85, 0.97)	0.954 (0.89, 0.98)
FaMSc ^8^	0.978 (0.95, 0.99)	0.978 (0.95, 0.99)
HC ^9^	0.979 (0.95, 0.99)	0.964 (0.91, 0.99)
HSc ^10^	0.88 (0.73, 0.95)	0.725 (0.43, 0.88)
**<0.5 poor** **0.5–0.75 good** **0.75–0.9 good** **>0.9 excellent**		

^1^ Upper arm lateral skin, ^2^ upper arm lateral subcutis, ^3^ upper arm medial skin, ^4^ upper arm medial subcutis, ^5^ forearm lateral skin, ^6^ forearm lateral subcutis, ^7^ forearm medial skin, ^8^ forearm medial subcutis, ^9^ hand skin, ^10^ hand subcutis.

**Table A2:** Limb centimetre measurements reliability testing.

Measuring Site	ICC (95% C.I.)	ICC (95% C.I.)
	Inter-Rater Reliability Researcher 1 and Researcher 1	Inter-Rater Reliability Researcher 1 and Researcher 1
Measuring point 0	0.999 (0.99, 0.99)	0.999 (0.99, 0.99)
Measuring point 1	1.00 (0.99, 1.00)	0.999 (0.99, 1.00)
Measuring point 2	1.00 (0.99, 1.00)	0.651 (0.41,0.81)
Measuring point 3	1.00 (0.99,1.00)	1.00 (0.99, 1.00)
