# Supplementary material for: High-Frequency Ultrasonography Imaging: Anatomical Measuring Site as Potential Clinical Marker for Early Identification of Breast Cancer-Related Lymphedema
Source: Biomedicines. 2025 Jun 6;13(6):1396. doi: 10.3390/biomedicines13061396 (PMC12191270; doi:10.3390/biomedicines13061396)
Supplement: Supplementary file 1 [file biomedicines-13-01396-s001.zip › biomedicines-3648906-supplementary.pdf]

Supplementary material

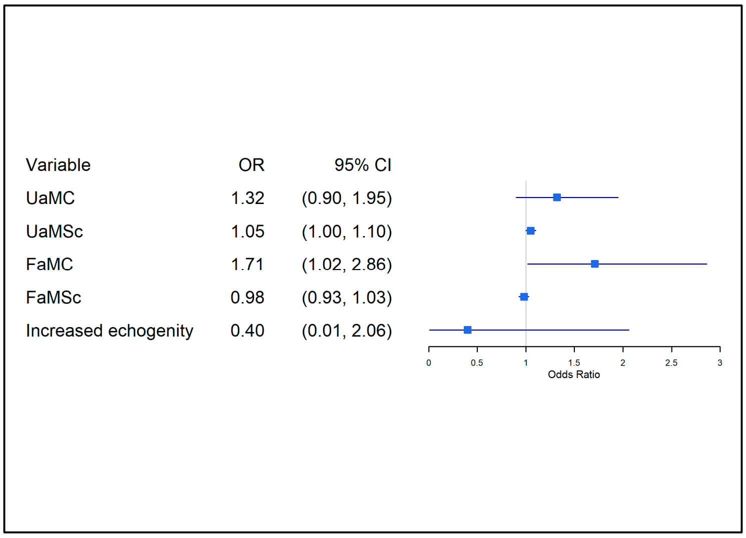

Figure S1. Forest plot showing summary results (Odds ratios (OR) and 95% confidence intervals (95% C.I) of multivariable regression analysis for the prediction of ISL stage. Multivariable model analysis revealed statistical significance for two variables: UaMSc and FaMC measuring points (* UaMC upper arm medial cutis, *UaMSc upper arm medial subcutis, *FaMC forearm medial cutis, *FaMSc forearm medial subcutis). Specifically, as the interlimb skin thickness increased in the FaMC and UaMSc regions, the likelihood of a higher ISL stage also increased (Table 4.).

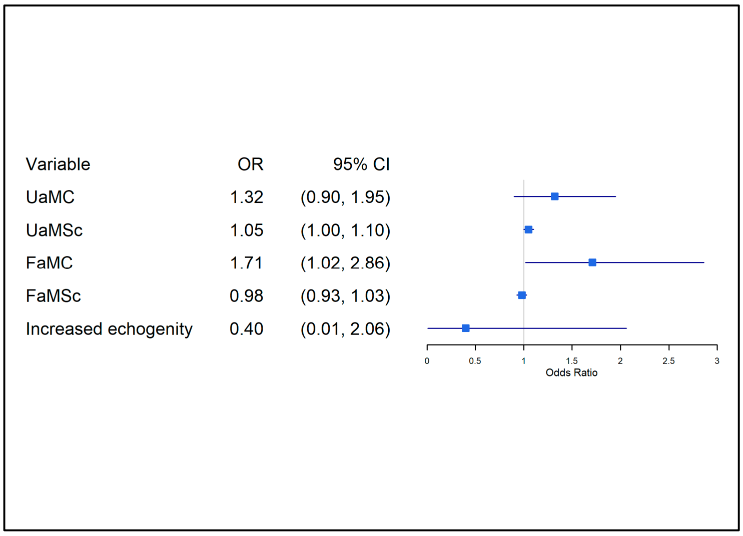

Figure S2. Forest plot showing summary results (Odds ratios (OR) and 95% confidence intervals (95% C.I) of multivariable regression analysis for the prediction of RVC 10%. Multivariable model analysis revealed statistical significance for one variable: FaMC measuring point (*UaMC upper arm medial cutis, *UaMSc upper arm medial subcutis, *FaMC forearm medial cutis, *FaMSc forearm medial subcutis). Specifically, as the interlimb skin thickness increased in the FaMC region, the likelihood of RVC 10% also increased (Table 5.).

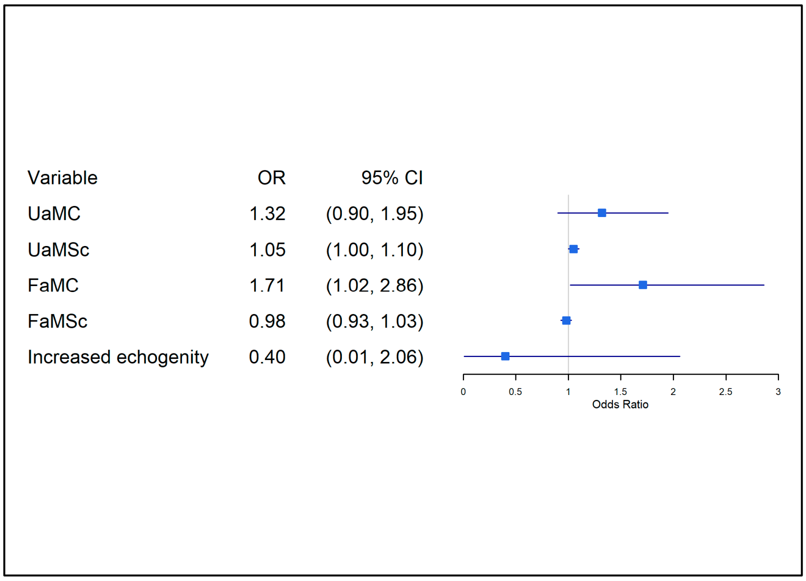

Figure S3. Forest plot showing summary results (Odds ratios (OR) and 95% confidence intervals (95% C.I) of multivariable regression analysis for the prediction of RVC 5%. Multivariable model analysis revealed statistical significance for one variable: UaMC measuring point (*UaMC upper arm medial cutis, *UaMSc upper arm medial subcutis, *FaMC forearm medial cutis, *FaMSc forearm medial subcutis). Specifically, as the interlimb skin thickness increased in the UaMC region, the likelihood of RVC 5% also increased (Table 6.)

Table S1. Results of the sensitivity analysis for the multivariate logistic regression analysis for the prediction of lymphedema relative volume change ≥5%. We included each of the variables—BMI, radiotherapy, and axillary surgery type—individually in the model

	**Multivariate model adjusted for age**		**Multivariate model adjusted for BMI**		**Multivariate model adjusted for RT**		**Multivariate model adjusted for axillary surgery**	
Variable	OR (95% C.I)	p-value	OR (95% C.I)	p-value	OR (95% C.I)	p-value	OR (95% C.I)	p-value
UaMC[3], median (IQR)	1.49 (1.01, 2.21)	**0.047**	1.54 (1.06,2.24)	**0.025**	1.55 (1.06, 2.26)	**0.024**	1.54 (1.05, 2.25)	**0.027**
UaMSc, median (IQR)	1.01 (0.98, 1.05)	0.408	1.02 (0.98, 1.06	0.395	1.01 (0.98, 1.05)	0.519	1.01 (0.98, 1.05	0.529
FaLSc[6], median (IQR)	1.09 (0.99, 1.21)	0.087	1.1 (0.99,1.22)	0.080	1.09 (0.99, 1.21)	0.080	1.09 (0.99, 1.21	0.089
FaMC[7], median (IQR)	1.23 (0.87, 1.73)	0.238	1.17 (0.85, 1.61)	0.337	1.21 (0.87, 1.69)	0.257	1.19 (0.87, 1.64	0.280
FaMSc[8], median (IQR)	1.03 (0.97, 1.19)	0.329	1.04 (0.98, 1.12)	0.221	1.04 (0.97, 1.10)	0.261	1.04 (0.97, 1.12)	0.239
Arm echogenity category n (%)		0.673		0.564		0.528		0.652
Increased echogenity	Reference		Reference		Reference		Reference	
Normal echogenity	0.71 (0.15, 3.48)		0.64 (0.14, 2.95)		0.60 (0.13, 2.91)		0.70 (0.15, 3.24)	
